# Differential effects of forest environmental perception on psychological well-being: the moderating role of perceived stress in national parks

**DOI:** 10.3389/fpubh.2026.1853526

**Published:** 2026-09-18

**Authors:** Weina Ni, Jing Li

**Affiliations:** 1 East China Normal University, Shanghai, China; 2 School of Management and Economics, University of Electronic Science and Technology of China, Chengdu, China; 3 ISCTE Business School, ISCTE Lisbon University Institute, Lisbon, Portugal

**Keywords:** forest environmental perception, moderation analysis, national parks, perceived stress, psychological well-being

## Abstract

**Introduction:**

The rapid urbanization and exposure to unnatural environments has helped to escalate global mental health problems. Interventions involving forests have been demonstrated to be therapeutic, but current studies are mostly concerned with direct effects, not taking into consideration environmental perception and individual differences. Specifically, the moderating effects of stress in determining the psychological well-being benefits of forest settings are not well studied. This study therefore examined whether perceived stress moderates the association between forest environmental perception and psychological well-being among adult visitors to a national park.

**Methods:**

This quantitative cross-sectional study involved 300 adult visitors to Zhangjiajie National Forest Park, China. Recruitment continued until 300 eligible, consenting visitors completed the questionnaire. Ineligibility and refusal were resolved at the point of approach, before enrolment, and every questionnaire collected passed an on-site completeness check, so 300 was both the number collected and the number analysed, with no case excluded at the analysis stage. Because recruitment used non-probability purposive intercept sampling, the findings describe this visitor sample rather than a defined population, and all interval estimates are model-based rather than design-based. Validated measures were used to collect data on forest environmental perception, perceived stress (PSS-10), and psychological well-being (WHO-5). All instruments demonstrated acceptable internal consistency reliability (Cronbach’s α > 0.70). SPSS and R were used to perform Pearson correlation and hierarchical regression analyses to test moderation, with the addition of an interaction term for forest environmental perception and stress.

**Results:**

Psychological well-being had a positive relationship with forest environmental perception (*r* = 0.61, *p* < 0.001), and perceived stress was negatively related to it (*r* = −0.55, *p* < 0.001). Regression analysis revealed that environmental perception had a significant and positive association with psychological well-being (*B* = 21.50, *p* < 0.001), whereas perceived stress was negatively associated (*B* = −1.53, *p* < 0.001). The interaction term was also significant (*B* = −1.29, *p* < 0.001), indicating that perceived stress statistically moderated this association, with the conditional slope falling from *B* = 29.88 at low stress to *B* = 13.12 at high stress. Perceived stress was unrelated to forest environmental perception itself (*r* = −0.05, *p* = 0.436). A significant amount of variance was explained by this model (*R*^2^ = 0.714).

**Discussion:**

Because all variables were measured concurrently, no causal or temporal ordering is implied. The results indicate that the psychological well-being gains of forest environments are not limited to environmental perception but also depend on personal stress levels. Notably, the attenuation observed at high stress does not arise from stressed visitors appraising the forest less favourably, but occurs downstream of appraisal—in the conversion of a favourable appraisal into felt well-being. This pattern is consistent with stress-related constraints on attentional capacity, ruminative self-focus, and emotion regulation. This research adds a moderation-based framework to the literature and underscores the necessity of individually tailored nature-based interventions. Accordingly, we propose a three-tier scheme for national park management and public health practice, in which self-directed access, guided restorative programming, and clinically partnered repeated-dose provision are matched to low, moderate, and high visitor stress, respectively.

## 1 Introduction

The recent surge in urbanization and technological development has radically reshaped human-environment relations, which have led to a significant increase in mental health issues in the world, such as stress, anxiety, and depression. Modern life, which is highly cognitive, sedentary and lacks physical interaction with the natural surroundings, has been uniformly associated with psychological distress and decreased emotional wellbeing. Due to this, a growing concern lies in determining non-pharmacological interventions that are sustainable and capable of improving psychological well-being outcomes as well as long-term well-being. In this environment, nature-based therapy, specifically exposure to forest settings, has become an encouraging and affordable way of promoting psychological well-being by directly engaging in natural ecosystems (1, 2).

Forest settings provide multisensory experiences that are rich and help to induce physiological relaxation and cognitive restoration. Theoretical models: Attention Restoration Theory and Stress Reduction Theory postulate that natural environments facilitate the restoration of mental fatigue through involuntary attention and facilitating emotional regulation activities. These theoretical points of view are highly empirically supported and indicate that exposure to forest environments can decrease such physiological indicators of stress, as cortisol levels and blood pressure, and, at the same time, alleviate mood states and psychological functioning (3, 4). Moreover, the availability of forest-derived volatile organic compounds (VOCs), including phytoncides, has been reported to affect immune responses and neurophysiological processes and increase the therapeutic benefits of forest ecosystem (5).

In the last 10 years, there has been an accumulating literature that has methodically explored the therapeutic value of forest therapy and nature exposure on psychological well-being. Current umbrella reviews and meta-analyses offer strong evidence that forest bathing (shinrin-yoku) has a significant positive effect on psychological well-being, alleviates the symptoms of anxiety and depression, and makes life, in general, more satisfying (6, 7). On the same note, experimental and observational research reports that even the shortest exposure to natural settings can provide significant changes in mood and stress reduction, even in people with psychological well-being issues, and even in 10 min (8). Moreover, comparative research shows that both outdoor and indoor green spaces have a positive effect on psychological well-being outcomes, but natural outdoor environments tend to have more robust and reliable effects (9, 10).

Regardless of these developments, new studies indicate that therapeutic impact of nature exposure is not universal and can be different with regard to environmental features and personal variation. Forests have structural characteristics such as biodiversity, vegetation density, and sensory attributes, such as sound and visual complexity, which are also critical in influencing psychological responses (11, 12). In addition, the recent advances in environmental neuroscience have offered more information about the mechanisms by which physical environments affect psychological well-being and the importance of cognitive appraisal and perceptual processing (13). This has resulted in the growth of academic interest in the topic of forest environmental perception, which referred to the subjective assessment of the environmental qualities of naturalness, safety, aesthetic value and tranquility (as assessed by individuals).

Growing body of evidence indicates the importance of environmental perception as a determinant of psychological benefits of nature exposure. Those who hold more positive views about forest settings are more likely to experience stress reduction and emotional enhancement than those who have less positive views (14, 15). Moreover, the existing research on green space exposure shows that positive environmental perception increases engagement, maladaptive thinking like rumination, and psychological resilience (16). These results highlight the fact that the therapeutic usefulness of forest settings goes beyond the physical exposure and is greatly permeated by subjective cognitive and emotional interpretations.

But one of the debilitating constraints of the current literature is that it does not focus enough on individual-level psychological variations, especially baseline levels of stress, in responding to nature-based interventions. Although the direct impacts of forest exposure on psychological well-being have been determined in a number of studies, very few studies have investigated the potential impacts of individual stress levels on these impacts. There is evidence that people who are highly stressed might be more or less responsive to nature-based therapy and can have more or less benefits because of the necessity to be restored or the effects of cognitive overload and poor perception (17). Moreover, certain studies may vary in terms of methodological heterogeneity, including the duration of exposure, environmental factors, and the characteristics of participants, making it further difficult to interpret the findings (18).

The evidence base is, however, considerably less uniform than the summary above suggests, and the disagreements within it are systematic rather than random. Three recurring points of conflict are worth setting out explicitly, because the present study is designed around them. The first concerns the magnitude and durability of benefit. Umbrella reviews of forest bathing report consistent improvement in mood and anxiety (19, 20), whereas meta-analyses using standardised mood instruments recover smaller pooled effects with substantial between-study heterogeneity (12, 21), and experimental work shows that exposures as short as 10 min produce detectable but short-lived change (8). The second concerns which environmental properties matter. Some meta-analyses identify forest structure and altitude as decisive (9, 22); others locate the operative factors in thermal and acoustic conditions (5, 23); and a further body of work finds that simulated and video-based nature reproduces a large share of the benefit, which implies that the physical attributes of a real forest are not strictly necessary (6, 7, 11). The third concerns for whom the benefit holds. Reviews of disadvantaged and clinical groups report amplified effects (8, 17), while reviews of nature prescriptions and outdoor physical activity report attenuated or inconsistent ones (24–26).

These inconsistencies share a structural feature that points directly to the gap this study addresses. Almost all are analysed as differences in the average effect between studies, rather than as conditional effects estimated within them. Where moderators are examined, they are overwhelmingly environmental or dose-related: Bolouki et al. (27) test duration, nature type, sample characteristics and immersiveness; Song et al. (23) test thermal conditions; Kim et al. (9) test altitude; Kim et al. (22) test forest structure. Each asks what property of the setting changes the benefit. Comparatively little work asks what property of the person changes it, and where psychological state does appear it is modelled as an outcome of exposure rather than as a condition upon it (2, 28). This matters because two theoretical accounts make directly opposing predictions about stress and have not been adjudicated against each other. A resource-need account holds that greater depletion leaves more room for recovery, so benefit should be larger among the highly stressed (19). A capacity-constraint account holds that restoration is not passively received but requires residual attentional and regulatory capacity, which stress erodes, so benefit should be smaller (29–31). The existing literature cites both without testing either. A second, narrower gap compounds this: forest environmental perception is almost always collapsed into a single composite, so it remains unknown which perceived attributes carry the association with well-being (13, 30).

The most recent developments in digital and virtual studying of nature also support the significance of perception and the differences between people. Research has shown that simulated nature scenes may cause quantifiable effects of psychological benefits but these results are usually tempered by realism, sensory integration, and user characteristics (8, 32). Although these results add to the understanding of the mechanisms of nature-based therapy, they also indicate the need for more ecologically valid, data-driven studies conducted in real-life settings, especially in highly protected natural areas, such as national parks.

In this respect, the Chinese national parks, especially those with high biodiversity and preserved forest ecosystems, such as Zhangjiajie National Forest Park, will be a perfect setting to examine the therapeutic implications of perceptions of the forest environment. These settings provide special ecological features, such as low anthropogenic activity, high plant diversity, and immersive natural experiences, and are therefore very appropriate to study the interdependence between the perception of the environment and the psychological consequences.

Although national parks are considered to be an important therapeutic landscape, the study gap still exists in the area of interaction between the environmental perception of forests and the individual stress levels and their effects on psychological well-being outcomes. The available literature is mostly concerned with direct relationships between exposure to nature and well-being with little regard to the moderating mechanisms that could provide explanations as to why different individuals react differently. It is crucial to fill in this gap to create more specific and individual nature-based interventions.

We know that nature exposure is linked to psychological well-being, but few studies have investigated the contribution of the perceived qualities of nature to psychological outcomes. Although many studies include several dimensions (such as naturalness, tranquility, safety, biodiversity richness and restorative quality) of forest environmental perception, they generally aggregate them into a single composite construct. Moreover, little consideration has been given to the relationship between individual psychological states and their respective strength in an actual national park context, especially to the extent of perceived stress. Filling these gaps could help provide a more complex understanding of the role of forest environments in supporting psychological well-being for whom and how.

Thus, the present study examines how the association between forest environmental perception and psychological well-being varies that can specifically be analyzed through the moderating role of an individual level of stress in national parks. This research has three objectives: (1) to test the connection between forest environmental perception and psychological well-being outcomes; (2) to test the moderating role of personal stress level on this connection; and (3) to test the interaction patterns that contribute to the difference in psychological responses to forest environments.

This study makes a both theoretical and practical contribution. Theoretically, it moves the current literature forward in the sense that the environmental perception and moderating stress is combined into a single analytical model, thus expanding the current models of nature-based therapy and environmental health. In practice, the results offer evidence-based information to policymakers, practitioners in the field of public health, and government parks managers to develop a more effective and individualized nature-based intervention. This study will help to create adaptive, sustainable and context-specific strategies to enhance psychological well-being using natural environments by highlighting the role of individual differences in determining therapy outcomes.

## 2 Method

### 2.1 Study design and setting

The selected analytical design was a quantitative cross-sectional one to test the hypothesis that forest environmental perception is related to psychological well-being, and to test a hypothesis whether individual stress levels can moderate the relationship in national parks. The cross-sectional design was deemed suitable since the main aim of the study was to determine the trends in relation to naturally occurring variables that were to be measured at a single point of time in the real environmental conditions (33). The design is common in the environment and population health studies where the aim is to determine the relationship between perceived environmental attributes and psychological outcomes in a specified sample.

The nature of the research question also had justification in the design. The current study was not aimed at experimentally manipulating the forest setting, but rather, at assessing the perceptions of individuals in the current forest settings and the relationship that exists between them and psychological well-being status, given different levels of stress. Since the perception of the environment is subjective and context-specific, data gathered in a natural environment under a national park setting involved a greater ecological validity in comparison to laboratory research or simulated exposure research. Moreover, the analysis model was created to go beyond mere association by evaluating an interaction effect between forest environmental perception and personal stress, which will offer a more complex perspective on the varying psychological well-being reactions.

The research was carried out in Zhangjiajie National Forest Park, Hunan Province, China, which is a preserved region that is high in biodiversity with dense forest cover and low urban interference. The place of the study was China because it has a large area of forests and it has been long established that they have been implementing nature-based therapeutic interventions, such as forest therapy and ecological health promotion programs. The Chinese national parks are ideal locations to study the effects of exposure to forests in health because they have maintained ecological integrity and well-organized management systems and are experiencing an influx of people into nature-related activities.

The chosen park is characterized by a variety of vegetation, such as mixed broadleaf and coniferous forest, natural soundscapes, shaded walking paths, and fairly low rates of anthropogenic noise. These environmental features are critical to the investigation of environmental perception of forests because they determine the sensory experience, perceived restoration and psychological feelings of people.

The data collection process was performed within 1 months (October, 2025) to provide the consistency in the environmental conditions as well as the availability of participants. The chosen period reduced seasonal differences in vegetation cover and climatic factors, thus increasing the validity of environmental exposure and perception measurements. The data were collected during the day, in favorable weather, to ensure consistency in participants’ experiences.

### 2.2 Participants and sampling

The population of interest was adult visitors to the national parks who had a direct encounter with the forest during their visit. The inclusion criteria were the following: the participants had to possess the following qualities:

- between 18 years of age and above;
- able to read and understand the study questionnaire language;
- was at least 20–30 min in the forest area before finishing the survey;
- gave informed consent.

The exposure threshold was kept at a minimal level to ensure that participants had sufficient exposure to the forest environment to make meaningful perceptual judgments. Those who were excluded were those who:

- had a standard age between 18 years and below;
- had not yet crossed or known the forest area;
- could not fill in the questionnaire on their own; or
- claimed that he had severe acute distress during the time of participation and was unsuitable to complete the survey.

The number of participants was aimed to be 300. This sample was deemed sufficient to conduct moderation analysis via multiple regression since it offers sufficient statistical power to identify small-moderate effects of interaction and also offers the ability to control demographic covariates. When direct effects of a study are larger than the moderation effects, a large sample is usually used to enhance stability of estimates and decrease chances of Type II error, particularly in research that deals with perception variables of psychology and environment. This size of sample is also sufficient to enhance subgroup representation on the levels of stress levels, gender and age. A formal sensitivity analysis supports this target. With n = 300, three predictors, alpha = 0.05 and powe*r* = 0.80, the design detects an incremental effect of *f*^2^ = 0.026 for the interaction term, that is, an effect below Cohen’s conventional threshold for a small effect (34). The interaction actually observed was substantially larger than this detection floor (Δ*R*^2^ = 0.061, *f*^2^ = 0.21; Section 4.4), so the study was not operating at the margin of its sensitivity.

Despite the collection of demographic data which was done in a descriptive way, the current analysis concentrated on the key research variables (forest environmental perception, perceived stress, and psychological well-being) to test the hypothesized moderation model.

A non-probability purposive intercept sampling technique was used. The visitors were approached at specific resting areas, trail exits, and visitor areas in the park after spending enough time in the forest setting and met the inclusion criteria. This method was chosen due to the fact that the research needed to have participants that were directly and in close proximity to the forest and not a general population sample. Field intercept sampling was also convenient and effective in finding qualified respondents in the naturalistic environment. This sampling method is usually adopted in field research in the environmental and public health area where the respondents should be those in close contact with the area under study.

The recruitment procedure was as follows. Fieldwork was carried out by trained research assistants stationed at the principal resting areas, trail exits and visitor areas of the park. Sessions were distributed across both weekdays and weekend days throughout the October 2025 collection window and across morning and afternoon periods, during daylight hours and in fair weather, so that recruitment was not clustered within any single day type or time of day. Within each session, assistants approached visitors in the order in which they passed the intercept point, rather than selecting them on the basis of appearance, behavior or apparent willingness to take part. Every visitor approached was screened verbally against the eligibility criteria listed above and given a standardised verbal and written account of the study’s purpose, its procedures and its voluntary nature before being invited to participate. Those who consented completed the questionnaire on site, and assistants remained available to answer questions about item wording without prompting any particular response.

Even though probability sampling might have been more representative of the population, purposive field-based sampling was used in this case since the research was limited to a situation specific experience: the perceived attributes of a forest setting during a visit to a national park. In order to minimize selection bias to the greatest extent possible, recruitment was done over various days of the week and also at various times of the day, and only eligible visitors were recruited in a systematic, as opposed to selective, manner (i.e., not based on appearance or behavior).

Recruitment continued until 300 eligible, consenting visitors had completed the questionnaire. Ineligibility and refusal were therefore both resolved at the point of approach, before enrolment: a visitor who did not meet the eligibility criteria, or who declined, was not enrolled and contributed no questionnaire. Every questionnaire that was collected was then checked for completeness on site before the respondent left the survey point, and all 300 were complete and were retained. The figure of 300 is accordingly both the number of questionnaires collected and the number analysed. No case was excluded after enrolment, and no analysis reported in Section 4 involves listwise deletion. Figure 1 sets out the flow.

**Figure 1:**
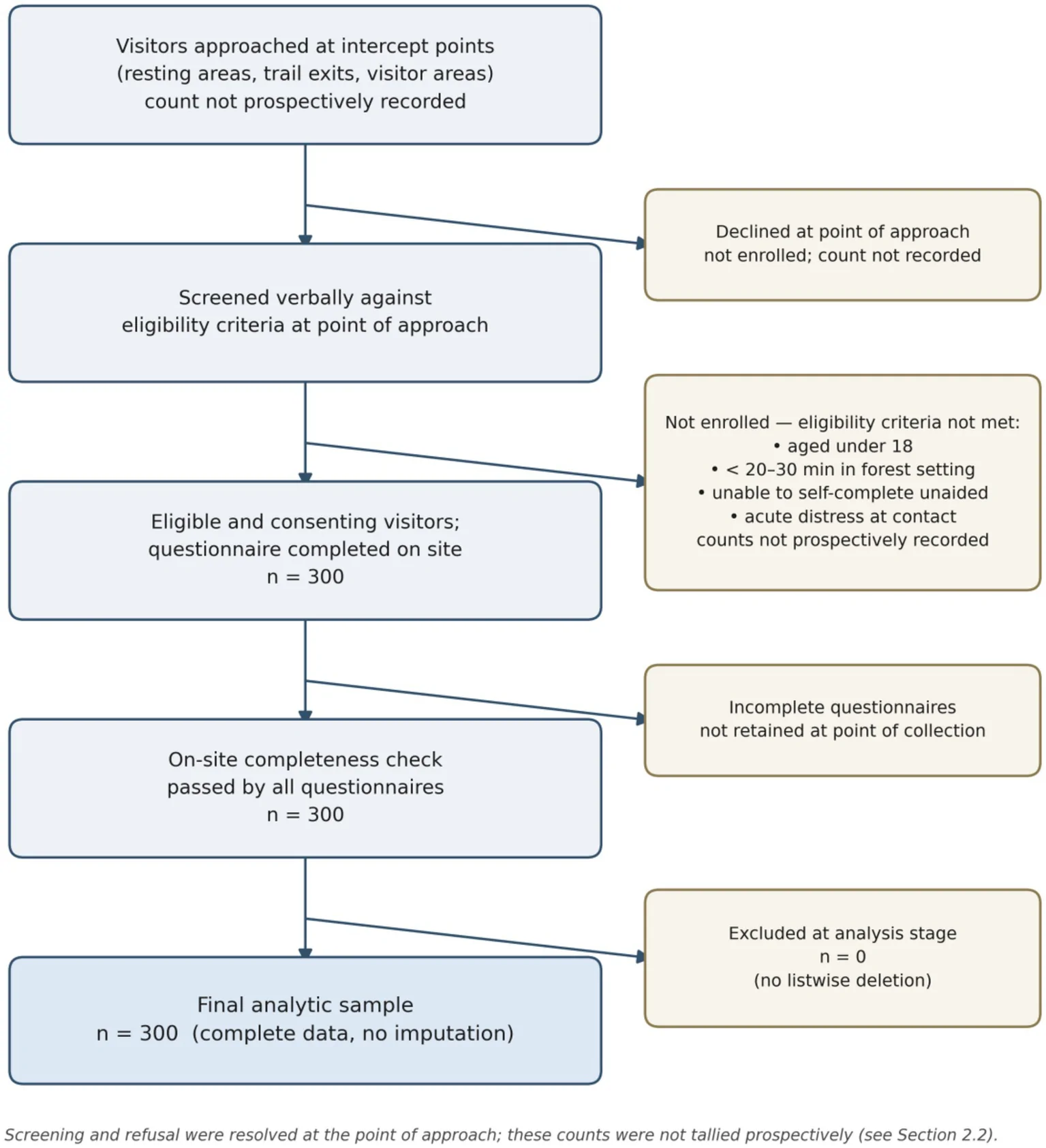
Participant flow from initial approach to final analytic sample. Recruitment continued until 300 eligible, consenting visitors had completed the questionnaire. Ineligibility and refusal were resolved at the point of approach, before enrolment, and were not tallied prospectively; a response rate therefore cannot be computed and this is recorded as a limitation in Section 5.5. All questionnaires collected passed the on-site completeness check, so *n* = 300 is both the number collected and the number analysed, with no case excluded at the analysis stage and no imputation performed.

One consequence of this procedure should be recorded frankly rather than glossed. Because eligibility screening and refusal were both handled conversationally at the point of approach, the study did not maintain a prospective running count of visitors approached, visitors declining, or exclusions by individual criterion. A refusal rate therefore cannot be computed, and we decline to estimate one retrospectively. This is a genuine limitation of the fieldwork design and is carried into Section 5.5: without a record of non-participants, the possibility that those who declined differed systematically from those who consented cannot be assessed. We note the corrective for future field studies of this design, since it is inexpensive and would have cost this study nothing to implement: every approach, refusal and screening exclusion should be tallied prospectively on a structured field log, which permits both a response rate and a formal assessment of non-response bias.

Two features of this sampling strategy qualify the interpretation of the findings. First, the sample rests on no probability-based sampling frame. The standard errors and confidence intervals reported in Section 4 are therefore model-based: they quantify uncertainty in the fitted regression parameters conditional on the realised sample under the usual ordinary least squares assumptions, and they do not represent design-based sampling error relative to any enumerated population. No estimate in this paper should be read as an estimate of a population parameter for Chinese national park visitors, for residents of Hunan Province, or for the Chinese adult population. Second, the scope of the sample is narrow and is best described narrowly: adults aged 18 years and over who visited Zhangjiajie National Forest Park during daylight hours in fair weather in October 2025, who had spent at least 20 to 30 min in forested areas before being approached, who were literate in the questionnaire language and able to self-complete it unaided, and who consented. By construction this excludes people who do not visit national parks at all, those for whom access is financially or physically prohibitive, those visiting in other seasons or weather conditions, and those in acute distress at the point of contact. The last of these deserves emphasis: because acutely distressed visitors were screened out, the upper tail of the perceived stress distribution is under-represented, and the moderation effect reported below is for that reason likely to be conservative rather than inflated. The findings should accordingly be read as conditional associations within a defined visitor sample, and any extension beyond that sample treated as a hypothesis for replication rather than as an inference.

### 2.3 Measures

The structured self-administered questionnaire was used to collect the data which included four sections: demographic characteristics, forest environmental perception, perceived stress, and psychological well-being outcomes. All scales were chosen based on previously validated scales or modified based on existing environmental psychology and psychological well-being scales.

#### 2.3.1 Forest environmental perception

The forest environmental perception scale was compiled for the present study by adapting items from established restorative-environment and environmental-perception instruments in environmental psychology. Its restorative-quality and tranquility dimensions follow the logic of the Perceived Restorativeness Scale (30), which operationalises the being-away, fascination, extent and compatibility components of Attention Restoration Theory (31); the remaining dimensions index perceived naturalness, visual attractiveness, safety, comfort and biodiversity richness, which recur across the environmental perception literature as the attributes through which visitors evaluate natural settings. Items were administered in Chinese. The resulting composite is an adapted instrument rather than a previously validated one, and we therefore rest no claim on the validation history of its source scales. The scale measured the subjective assessment of the participants about the forest in the dimensions of:

- perceived naturalness
- visual attractiveness
- tranquility
- safety
- comfort
- biodiversity richness
- restorative quality

Items were scored on a 5-point likert scale of 1 = strongly disagree to 5 = strongly agree. The scale had high reliability of internal consistency (Cronbach’s α = 0.89). Construct validity was also established using exploratory factor analysis of the scale. The response of items was averaged to obtain a composite score, and the score of a higher level of forest environmental perception was taken as positive.

This construct was the focus of the study since the research question did not focus on physical exposure to a forest, but instead, how participants cognitively and emotionally perceived the exposure. A multidimensional perception scale enabled the study to capture beyond scenic appreciation and represent the broader therapeutic attributes of forest environments.

Because the composite was assembled for this study, its psychometric behavior in the present sample was examined before it was entered into any inferential model. Internal consistency was good (Cronbach’s α = 0.89), and exploratory factor analysis supported the intended multidimensional structure rather than a single undifferentiated factor. The dimension-level correlations in Table 1 are consistent with that structure, since they diverge substantially from one another rather than moving together as a single construct would. Two qualifications follow, and we state both rather than either. First, this is construct evidence internal to the present sample: the instrument has not been validated against an independent sample or against external criteria, and Section 5.5 records this as a limitation on the strength of the measurement claim. Second, because the composite is adapted rather than standard, the absolute value of its mean is not comparable with values reported in studies using different instruments; only the associations estimated within this sample are interpretable. Item-level factor loadings and dimension-level reliability coefficients are available from the corresponding author on reasonable request.

**Table 1:** Correlations between forest perception dimensions, psychological well-being, and perceived stress.

Forest perception dimension	WHO-5 (*r*)	PSS-10 (r)
Perceived naturalness	0.48***	−0.21**
Visual attractiveness	0.52***	−0.24**
Tranquility	0.59***	−0.31***
Safety	0.46***	−0.19**
Comfort	0.50***	−0.26***
Biodiversity richness	0.43***	−0.18**
Restorative quality	0.63***	−0.35***

WHO-5, Psychological well-being; PSS-10, Perceived Stress Scale. *p < 0.05, **p < 0.01, ***p < 0.001.

#### 2.3.2 Individual stress levels

The Perceived Stress Scale (PSS-10) was used to measure stress; it is a psychometrically validated and widely used instrument measuring the degree to which people appraise life situations as stressful (35), administered here in its established Chinese-language form. The PSS-10 has 10 items that are rated on a scale of 5 where 0 = never, and 4 = very often. Following reverse-coding the positively stated items, the total amount of the responses was added to achieve a total stress score, and larger scores indicated more perceived stress.

PSS-10 was chosen since it measures subjective stress appraisal as opposed to exposure to external stressors alone, and thus it is very convenient in this study to be moderated. A proven global measure of stress should have been used as the hypothesis was about whether individuals with varying levels of stress derive varied psychological well-being benefits of forest environmental perception.

#### 2.3.3 Psychological well-being outcomes

The World Health Organization Well-Being Index (WHO-5) was used as a measure of psychological well-being and is a validated instrument comprising five items rated on a 6-point Likert scale, with well-documented clinimetric validity across settings and languages (36). The WHO-5 is a measure of positive psychological well-being, and has been extensively used in both public health and psychological well-being research due to its high reliability, conciseness, and sensitivity to changes in emotional well-being. The total of the items scores were transformed and transformed according to the standard scoring scales and the better the score the higher the psychological well-being condition and the psychological wellbeing. One property of this instrument should be noted at the outset because it bears directly on interpretation: the WHO-5 asks respondents about the preceding 2 weeks, so it indexes consolidated recent well-being rather than momentary state at the time of the visit. Section 5.5 discusses what follows from this for how the central association may be read.

The choice of a validated psychological well-being instrument was imperative to provide reliability, comparability with previous literature and construct validity. As the purpose of this article is psychological well-being within the context of national park therapy value, the use of a standardized and globally accepted measure contributes to the scientific rigor and reproducibility.

### 2.4 Pilot testing and instrument quality

The questionnaire was piloted before the main data collection began. A pilot group of 20 to 30 visitors, meeting the same eligibility criteria as the main study, completed the instrument under field conditions at the same intercept points shortly before the main collection window opened. The pilot assessed clarity of wording, comprehension of individual items, usability of the response format, typical time to completion, and the practical feasibility of administering the instrument in a field setting where respondents complete it standing or seated outdoors. On the basis of the pilot, minor rewording was applied to individual items of the forest environmental perception scale to improve readability; no item was added or removed, and no change altered the substantive meaning of any item, so the piloted and final versions are equivalent in content. The PSS-10 and WHO-5 were administered in their established Chinese-language forms and were not modified in any respect. Pilot responses were used solely for instrument refinement and form no part of the analytic dataset. Cronbach’s alpha was used to assess the internal consistency reliability of the study instruments. Cronbach’s alphas for each scale in the study ranged from 0.78 to 0.91, which is considered as acceptable to excellent internal consistency reliability. Construct validity was achieved through the use of validated instruments and the judgement of experts in the areas of public health, psychology, and environmental research. Moreover, exploratory factor analysis (EFA) was applied to analyze the structure of the forest environmental perception scale, which also confirmed that forest environmental perception is a multi-dimensional scale. To ensure the reliability, validity and accuracy of the questionnaire prior to final data collection, the following procedures were used.

#### 2.4.1 Variables and measurements

The study included three categories of variables.

#### 2.4.2 Independent variable

The continuous composite score obtained from items with a 5-point Likert-scale is referred to as forest environmental perception. The greater the value the more positive evaluation of the environment.

#### 2.4.3 Dependent variable

The selected validated instrument was used to compute one or more continuous score for psychological well-being, which was the dependent variable. Higher WHO-5 scores reflect better psychological well-being.

#### 2.4.4 Moderating variable

The individual stress level which is the moderating variable contains the PSS-10 total score, which is a continuous variable. For the purposes of description, stress may also be classified as low, moderate or high, yet the moderation analysis by regression was carried out on the continuous form to maintain information and power.

#### 2.4.5 Covariates

Demographic variables were gathered for descriptive purposes, yet excluded from the final regression model as this involved testing the hypothesized moderation relationship among the study variables.

### 2.5 Data collection

Asking personal eligibility inquiries were trained research assistants. The study was explained to participants and they were informed participation was voluntary, anonymous and not affiliated with the park service. Those who agreed completed a written informed consent before receiving the questionnaire.

The participants having spent minimal time in the forest setting completed the survey. The timing of the study was relevant as researchers aimed at capturing the immediate perception of the actual environment. Research assistants were available to answer procedural queries but not to suggest answers. All filled questionnaires were checked on site for completeness, wherever required, when not recorded identifying.

This anonymous minimal-risk questionnaire study obtained formal ethics exemption from the Human Subjects Protection Committee of East China Normal University. No clinical biological samples, identifiable personal information or sensitive private data were collected from participants. All participants were fully informed of the study’s purpose, anonymous data processing and their right to decline participation prior to completing surveys, and verbal informed consent was obtained from every respondent. All study procedures followed the ethical principles outlined in the Declaration of Helsinki. A copy of the official ethics exemption document can be provided upon journal editorial request.

### 2.6 Statistical analysis

The researchers entered data into IBM SPSS Statistics version 26 and checked, before running analysis, ensuring the integrity of the data. R was additionally used for robustness checks and for interaction visualisation. The procedures of data cleaning were checking for duplicate entries, missing values, inconsistent responses and outlier. Missing data require comparatively little discussion in this study, because the field procedure was designed to prevent them rather than to repair them afterwards. Every questionnaire was checked for completeness on site, before the respondent left the survey point, and any omitted item was drawn to the respondent’s attention at that moment so that it could be completed if they wished. Questionnaires that remained incomplete were not retained and did not enter the dataset. The 300 cases forming the analytic sample therefore contain no missing values on any study variable. No imputation of any kind was performed, no substitution rule was applied, no case was excluded at the analysis stage, and all 300 cases contributed complete data to every model reported in Section 4, so that n = 300 throughout and no reported statistic rests on a reduced subsample.

First, descriptive statistics were calculated for all study variables. Frequencies and percentages were reported for the categorical variables and means and standard deviations of constant variables. Skewness, kurtosis, histograms, and normal probability plots were used to test normality of continuous variables.

Pearson correlation analysis for continuous measures was used to test bivariate correlation. The final analysis did not include comparisons by demographic variables since there was no focus on this particular issue. To determine if there was any common method bias that could have arisen from self-reported cross sectional data, Harman’s single-factor test was performed through exploratory factor analysis (37). The findings from Harman’s test for a single factor revealed that the first unrotated factor accounted for 34.7% of the total variation, suggesting that common method variance was not a significant threat to the results. We treat this as a screening diagnostic only. Harman’s test is widely acknowledged to be insensitive, and a low first-factor loading indicates the absence of gross evidence for method bias rather than its demonstrated absence (37); this qualification is carried forward into the limitations.

A hierarchical multiple regression analysis was conducted to test the hypotheses.

Initially, the major predictor and moderator variables were introduced into the model.

In the second stage, after adding the interaction term for the forest environment perception and stress, the moderating effect was tested.

To minimize multicollinearity and enhance interpretation, the predictor and moderator variables were centered before forming the interaction term. The effect of forest environmental perception on psychological well-being varied according to the stress level demonstrated significant interaction coefficient. Where the interaction term reached significance, conditional (simple) slopes were computed at the mean and at one standard deviation above and below the mean of perceived stress, following Aiken and West (38), and plotted to describe the form of the interaction. These conditional slopes are model-implied estimates derived from the fitted regression, not estimates obtained from separately analysed subgroups.

Tolerances and variance inflation factors (VIFs) were used to assess multicollinearity. Tolerance values greater than 0.20 and VIF values below 5 indicated that multicollinearity was not a serious concern. Check model assumptions of linearity, homoscedasticity, independence of errors and normality of residuals for final interpretation. Results were considered statistically significant at *p* < 0.05. The findings included reporting of effect size or standardized beta coefficient to check their practical significance. Ninety-five per cent confidence intervals were computed for every regression coefficient and are reported alongside the point estimates. Consistent with Section 2.2, these are model-based interval estimates for the fitted parameters and are interpreted as such rather than as design-based intervals for population quantities.

Justification for this analytical strategy was based on the fact that moderation analysis is consistent with the study conceptual model. It does not presume that all people get the same psychological well-being benefit from perceiving forest environments. The study can test whether this depends on the participants’ existing stress levels. Consequently, a more clinically and theoretically meaningful interpretation can be achieved, consistent with the study’s aims to identify differential therapeutic effects in national park environments.

## 3 Conceptual framework and hypotheses

The current study is based on environmental health and psychological restoration theories, which suggest that natural environments promote psychological well-being both physiologically and cognitively. Conversely, the existing research hypothesizes that perceptions of forest environmental quality can influence psychological outcomes even in the absence of direct exposure (39). People tend to have an ecological sense of natural environments like naturalness safety and restorative quality that affect their emotional and cognitive response. In this context, the study proposes that different individuals will have different impacts of forest environmental perception on psychological well-being and the level of impact will depend on one’s stress level. Two accounts make opposing predictions at this point. A resource-need account holds that people under greater stress have more depleted resources and therefore more room for recovery, so that a favourable environmental appraisal should yield a larger well-being gain among them (19). A capacity-constraint account holds the opposite: restoration is not passively received but requires residual attentional and regulatory capacity to disengage from internal concerns and take up the restorative affordances of the setting (30, 31). Elevated stress erodes precisely that capacity - through impaired prefrontal control of attention (29), through perseverative self-referential thought that competes for the same attentional resources (40, 41), and through reduced capacity to convert a favourable appraisal into sustained positive affect (42). On this account the same appraisal should translate into a smaller well-being gain among the highly stressed. The present study is structured to distinguish these predictions empirically. Consequently, stress is defined as a moderating variable which to a certain extent changes the strength and direction of environmental perception and psychological well-being.

As such, the research framework (Figure 2) used for this study establishes forest environmental perception as an independent variable, psychological well-being as a dependent variable, and the stress level of an individual as a moderating variable while controlling the demographic features of age, gender, and exposure duration.

**Figure 2:**
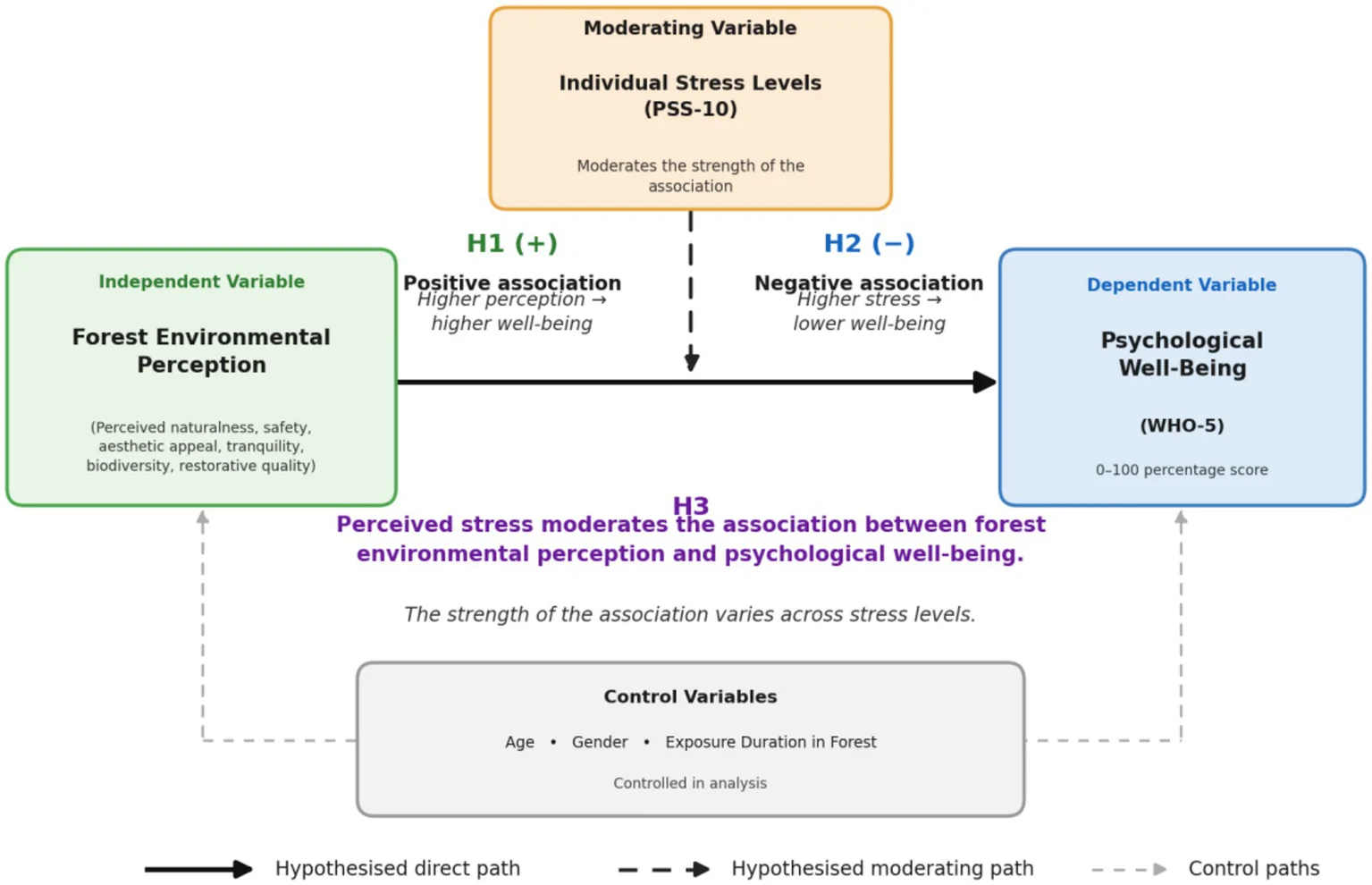
Hypothetical model that presents the perception of the forest environment to be a predictor of psychological well-being, with the perceived stress being a moderating factor. The model specifies a positive association of environmental perception (H1), a negative association of perceived stress (H2), and moderation of the first association by perceived stress (H3).

### 3.1 Hypotheses development

The ensuing hypotheses are evidenced by theoretical and empirical evidence.

*H1:* There is a positive association between forest environmental perception and psychological well-being. People who consider forest environments to be more restorative, safe and aesthetically pleasing will experience enhanced psychological well-being.

*H2:* Psychological well-being suffers when individuals feel elevated stress levels. There will be lower psychological well-being with higher perceived stress.

*H3:* The relationship between forest environmental perception and psychological well-being is significantly moderated by individual stress levels. Specifically, and consistent with the capacity-constraint account set out above, the positive association is expected to be weaker at higher levels of perceived stress. The degree of relationship’s strength between environmental perception and psychological well-being varies by the level of stress.

### 3.2 Statistical model

A moderation regression model was specified to test the hypotheses that were proposed.

Psychological well-being can be measured as a function of the perception of forest environment and stress level as follows:

Where.

- *β*₁ indicates direct impact of perception on forest environment.
- *β*₂ illustrates the impact of stress level.
- *β*₃ depicts the interaction (moderating effect)
- The error term is denoted by ε.

A statistically significant interaction term (*β*₃) suggests that environmental perception-psychological well-being links vary as a function of stress level.

## 4 Results

### 4.1 Descriptive statistics

A final analysis consisting of 300 observation was executed. Table 2 illustrates descriptive statistics (mean and standard deviation) for the principal study variables. Participants reported a relatively high level of forest environmental perception (*M* = 4.10, SD = 0.51). The sample had a mean perceived stress score of 18.20 (SD = 6.52) which indicates moderate stress. The average psychological well-being score of the respondents based on WHO-5 was 43.61 (SD = 18.10) which reflects moderately fine. The findings have enough variation on all three study variables to move ahead with the correlation and moderation analyses.

**Table 2:** Descriptive statistics of the study variables.

Variable	Mean	SD	Minimum	Maximum
Forest Environmental Perception	4.10	0.51	2.30	5.00
Perceived Stress (PSS-10)	18.20	6.52	0.00	40.00
Psychological well-being (WHO-5)	43.61	18.10	0.00	100.00

### 4.2 Correlation analysis

Forest environmental perception, perceived stress, and psychological well-being were subjected to Pearson correlation analysis for their bivariate relationships. As indicated in Table 3, there was a positive and significant correlation between forest environmental perception and psychological well-being (*r* = 0.61, *p* < 0.001), meaning the more one evaluated the forest environment positively, the better off they were mentally. Perceived stress showed a statistically significant negative correlation with psychological well-being (*r* = −0.55, *p* < 0.001), meaning that the higher the level of perceived stress, the poorer the psychological well-being.

**Table 3:** Correlation matrix of study variables.

Variable	1	2	3
1. Forest environmental perception	1.00		
2. Perceived stress (PSS-10)	−0.05	1.00	
3. Psychological well-being (WHO-5)	0.61***	−0.55***	1.00

***p < 0.001.

On the contrary, the association between forest environmental perception and perceived stress was negligible and not statistically significant (*r* = −0.05, *p* = 0.436). The implication of the finding is that positive perception of the environment and stress does not reflect heavily on each other in this dataset. There is no strong correlation of either of the two predictors so this is methodologically good as this would not cause any multicollinearity issues in the moderation model. The findings are shown in Table 3. This near-zero association is more than a multicollinearity check; it constrains how the moderation reported in Section 4.4 can be interpreted. It indicates that visitors reporting higher perceived stress did not appraise the forest environment any less favourably than those reporting lower stress. Whatever produces the attenuation of the perception-well-being association at high stress, therefore, it cannot be a difference in how the environment was perceived, and must instead concern what that perception yields.

### 4.3 Correlations between Forest perception dimensions and psychological well-being

Pearson correlation analyses (Table 1) were carried out among individual forest environmental perception dimensions, psychological well-being (WHO-5) scale, and perceived stress (PSS-10) scale to give a deeper insight into the nature of relationships between each specific forest environmental perception dimension and psychological outcomes. It was found that the restorative quality and tranquility variables showed the most significant positive correlations with psychological well-being, while the variable of perceived stress showed negative correlations with all forest environmental perception dimensions.

The highest positive correlation with psychological well-being was found in relation to the quality of restoration (*r* = 0.63, *p* < 0.001), while second highest positive correlation was found between tranquility and psychological well-being (*r* = 0.59, *p* < 0.001). Perceived stress was found to have a moderate negative relationship with both the quality of restoration and tranquility, which indicates that stressed individuals perceive forest environment to be less restorative.

### 4.4 Moderation analysis

To determine whether individual stress levels moderated forest environmental perception and psychological well-being relationship, an OLS regression model was estimated which included a centered interaction term. Table 4 shows the results. Forest environmental perception was positively and significantly associated with psychological well-being (*B* = 21.50, 95% CI [19.34, 23.66], *p* < 0.001). That is, more positive forest environmental perception is associated with a higher WHO-5 score. Perceived stress was negatively associated with psychological well-being (*B* = −1.53, 95% CI [−1.71, −1.35], *p* < 0.001). Above all, the interaction between forest environmental perception and perceived stress was statistically significant and negative (*B* = −1.29, 95% CI [−1.60, −0.98], *p* < 0.001), indicating statistical moderation of the association. Because forest environmental perception, perceived stress and psychological well-being were all measured concurrently in a single administration, this is a conditional association and not evidence of a causal moderating process operating during the visit.

**Table 4:** Moderation regression results predicting psychological well-being.

Predictor	*B*	*SE*	*β*	*t*	*p*
Constant	43.42	0.56	—	77.24	<0.001
Forest Environmental Perception (centered)	21.50	1.10	0.61	19.55	<0.001
Perceived Stress (centered)	−1.53	0.09	−0.55	−17.61	<0.001
FEP × Stress	−1.29	0.16	−0.24	−7.92	<0.001

Model summary: *R*^2^ = 0.714, Adjusted *R*^2^ = 0.711, *F* (3, 296) = 246.30, *p* < 0.001 Model-based 95% confidence intervals: Forest Environmental Perception [19.34, 23.66]; Perceived Stress [−1.71, −1.35]; FEP × Stress [−1.60, −0.98]; Constant [42.32, 44.52]. Incremental variance attributable to the interaction term: Δ*R*^2^ = 0.061 (*f*^2^ = 0.21). Intervals are conditional on the fitted model and on the realised non-probability sample (Section 2.2), and are not design-based estimates for a population parameter.

The regression model was significant and explained an overall variance of 71.4% for psychological well-being with *R*^2^ = 0.714; adjusted *R*^2^ = 0.711; *f* (3, 296) = 246.30; *p* < 0.001. Multicollinearity tests have produced satisfactory figures (VIF < 5; Tolerance>0.20), showing that multicollinearity was not a major concern. According to Harman’s single-factor test, the first factor accounted for less than 50 percent of the total variance, thus implying low common method variance. The interaction term accounted for a further 6.1% of variance in psychological well-being beyond the two constituent predictors (Δ*R*^2^ = 0.061, *f*^2^ = 0.21), which is a medium effect by Cohen’s conventions and well above the study’s detection floor reported in Section 2.2.

In terms of standardization, forest environmental perception has a positive effect (*β* = 0.61), perceived stress has a negative effect (*β* = −0.55), and the interaction term also remains significant (*β* = −0.24). The positive association between forest environmental perception and psychological well-being is progressively weaker at higher levels of perceived stress. Table 4 summarises the full model results.

#### 4.4.1 Interpretation of the interaction effect

To aid interpretation of the moderation effect, simple slope estimates were calculated at low stress (−1 SD), mean stress and high stress (+1 SD). The findings of the study revealed that with a low stress level, the positive slope of forest environmental perception on psychological well-being was the strongest (*B* = 29.88). Meanwhile, at the average level of stress, it had a moderate score (*B* = 21.50). At high stress the conditional slope was substantially smaller but remained positive (*B* = 13.12). These are model-implied conditional slopes derived from the fitted regression rather than estimates from separately analysed subgroups, and they are plotted in Figure 3 across ±1 SD of forest environmental perception.

**Figure 3:**
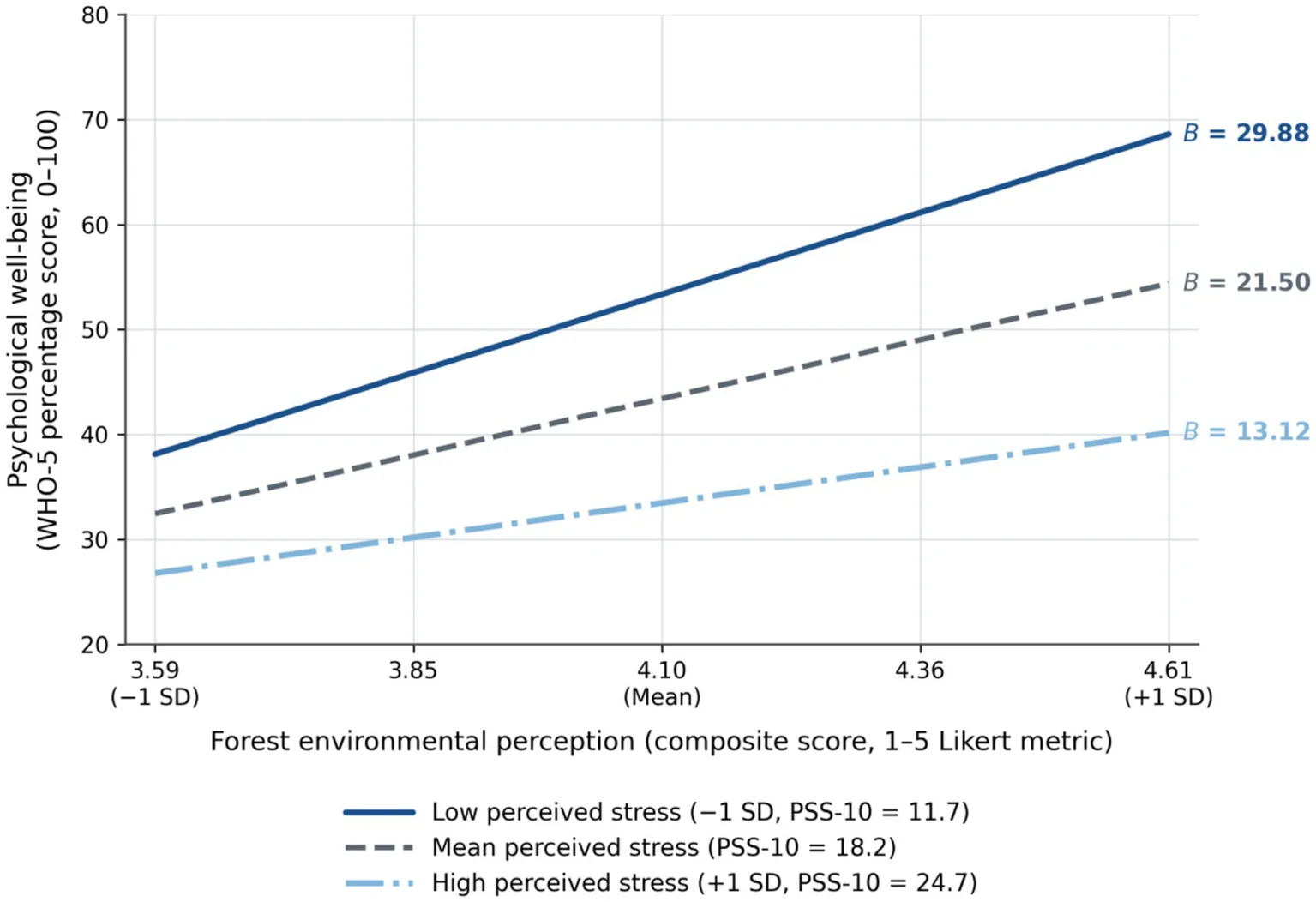
Conditional (simple) slopes for the association between forest environmental perception and psychological well-being at low (−1 *SD*), mean, and high (+1 *SD*) levels of perceived stress. The horizontal axis shows forest environmental perception as observed composite scores on the 1–5 Likert metric, plotted across ±1 *SD* around the sample mean (*M* = 4.10, *SD* = 0.51); the vertical axis shows psychological well-being as WHO-5 percentage scores on the full 0–100 scale. Lines are model-implied values from the moderation regression in Table 4 (constant = 43.42, *B* = 21.50 for forest environmental perception, *B* = −1.53 for perceived stress, *B* = −1.29 for the interaction). Low and high perceived stress correspond to PSS-10 totals of 11.7 and 24.7, respectively, (*M* = 18.20, *SD* = 6.52). The conditional slope is annotated at the right-hand end of each line. Note that all three slopes remain positive; the interaction attenuates the association rather than eliminating or reversing it.

Taken together, these estimates indicate that a more favourable appraisal of the forest environment is associated with higher psychological well-being at every level of perceived stress examined, but that the magnitude of this association falls by more than half between low and high stress. The association is attenuated among highly stressed visitors rather than absent, and we are careful not to overstate it in either direction. In effect, individuals that experience less stress can convert the positive impressions of their surroundings into greater well-being, whereas people with higher levels of stress receive lesser restorative value. The hypothesis that has been presented in this paper is that stress is a moderating variable which helps to determine the degree to which forest environmental perception can lead to psychological well-being.

The moderation effect (Figure 3) demonstrates that the positive correlation between forest environmental perception and psychological well-being is reduced with the increase in perceived stress.

### 4.5 Summary of hypothesis testing

Overall, the findings of the empirical research support the conceptual model. Psychological well-being is positively related to the forest environmental perception; thus, the first hypothesis was supported. Similar to the initial one, the second hypothesis was also validated, with perceived stress being negatively related to psychological well-being. Finally, the third hypothesis was supported: perceived stress statistically moderated the association between forest environmental perception and psychological well-being, and did so in the direction predicted by the capacity-constraint account rather than the resource-need account set out in Section 3. Collectively, these results are consistent with a model in which environmental appraisal and the individual’s psychological state jointly condition well-being in forest environments. As the design is cross-sectional, support for the conceptual model should be understood as the observed pattern being compatible with it, rather than as confirmation of the directional relationships it specifies.

## 5 Discussion

### 5.1 Forest environmental perception and psychological well-being

The current study investigated the correlation between forest environment perception and psychological well-being in a national park context, with particular reference to the moderation effect of individual stress. The results reveal three key trends. The forest environment was positively related to psychological well-being, meaning those who evaluated the forest environment as more restorative, safe, beautiful, and calming reported better psychological well-being. The second association was that perceived stress was negatively associated with psychological well-being. Even among visitors in a highly restorative natural setting, higher perceived stress accompanied markedly lower reported well-being. Most importantly, stress significantly moderated the association between forest environmental perception and psychological well-being. Specifically, the positive association with environmental perception was weaker at higher levels of perceived stress. Taken together, these findings affirm the conceptual model of this study and provide additional evidence that the psychological benefits of forest settings happen due to two main factors: not just the exposure but also subjective appraisal and individual psychological condition (43). We frame all three findings as associations throughout what follows. The study measured forest environmental perception, perceived stress and psychological well-being at a single point in time, and no design element permits the direction of any of these relationships to be established.

The positive correlation discovered between the perception of forest environment and psychological well-being reinforces and corroborates the growing body of evidence that the therapeutic value of nature is believed to stem more from the perception of the environment rather than the mere presence in greenery. Forest bathing and related nature-based interventions have been shown to improve mood and reduce anxiety and enhance well-being when environments are experienced as restorative and emotionally supportive (44). Similarly, nature exposure, in general, the psychological benefits are greatest when natural areas convey an attractive, safe and calming set of features (28). Findings from the present study extend this literature by demonstrating that the influence of ecological settings on psychological well-being can be accounted for by the forest perception in a real-life national park. The health value of national parks may not only be ecological or recreational, but also perceptual and experiential (45).

The specific characteristics of forests that lead to therapeutic effects have been studied before. The therapeutic effects of forest structures, biodiversity and spatial compositions can vary (50). Tranquility and sensory integration of the environment are significant (5). In the present study, even though forest structure was not objectively measured as a predictor, the perception measure indirectly captured participants’ appraisal of these environmental attributes. Perhaps this explains why the perception of the environment positively relates to psychological well-being. The finding also dovetails with recent work suggesting that environmental neuroscience can explain how external ecological features are translated into internal psychological states through perceptual processing and appraisal (23). In that regard, the present research affirms a structurally mediated understanding of natural therapy in which environmental benefit is not automatic but filtered through people’s understanding.

### 5.2 Moderating role of perceived stress

It is not surprising that perceived stress is negatively associated with psychological well-being, but its strength is noteworthy. Recent studies reiterate the established understanding within public health and mental health literature that stress is one of the strongest predictors of lower psychological well-being. A review of green prescriptions, greenspace interventions, and forest therapy all show that natural settings can diminish stress, though they will not erase an existing psychological burden (27).

The present findings are thus important because they suggest that experience of poorer psychological well-being is also a phenomenon faced by those with high stress levels even in the context of a highly restorative environment. Nature-based exposure may not be a sufficient stand-alone intervention to alleviate stress in highly stressed people, suggesting other factors. It might rather work best as part of a wider strategy for psychological well-being support.

The novelty of the study lies in the moderation effect. The result that the positive link between forest environmental perception and psychological well-being weakens at higher levels of stress means that stress constrains the extent to which people can psychologically gain from restorative environments. This finding is a potential valuable addition to the literature. Various studies in the past suggest that individuals experiencing greater stress should benefit more from nature since they have a stronger need for restoration.

Some studies showing how nature prescriptions as well as forest interventions may reduce stress and enhance emotional outcomes for vulnerable groups (13) lend some support for this interpretation. But study findings now indicate that high baseline stress may also undermine the capacity to cognitively and emotionally engage with restorative features of the environment. According to the analysis, distressed people entering the forest may already have reduced attentional flexibility, emotional receptivity, or cognitive rumination. Thus, they are not able to reap the therapy value of the place.

#### 5.2.1 Why elevated perceived stress may constrain restorative benefit: candidate mechanisms

This view is in line with restoration theory. Attention Restoration Theory holds that natural environments support recovery by engaging involuntary attention through soft fascination, allowing the directed-attention system that everyday demands deplete to rest and recover (31, 46); systematic review evidence for the attentional component is positive but modest and heterogeneous across populations (21, 47). The restorative process may not function as efficiently in the event that a person is already suffering from overwhelming internal tension, intrusive thought patterns, and/or emotional flooding. Stress Reduction Theory proposes a complementary and largely pre-cognitive route, in which an unthreatening natural setting elicits a rapid affective and psychophysiological shift towards recovery (48). That initial response, however, is attenuated where the person remains cognitively engaged with the stressor (20). Present results provide a more elaborate contribution to both nature and restoration framework that the internal psychological state is not only an outcome of nature exposure but also a condition in which the restorative process will take place. This is an important theoretical contribution because it shifts the discussion from does nature help with psychological well-being to analytical scales of nature’s impact. Under which psychological circumstances does nature impact psychological well-being most positively?”

Because the negative moderation is the principal novel contribution of this study, it warrants a more specific mechanistic account than a general appeal to restoration theory can provide. The starting point is an empirical constraint supplied by the present data, and it is a useful one because it eliminates the most obvious explanation. Perceived stress was essentially unrelated to forest environmental perception (*r* = −0.05, *p* = 0.436): highly stressed visitors did not rate the forest as less natural, less tranquil, less safe or less restorative than their less stressed counterparts. Whatever produces the attenuation, therefore, it does not operate by degrading perception of the environment. It operates downstream of perception, on the conversion of a favourable environmental appraisal into felt psychological well-being. This narrows the field of plausible mechanisms considerably. Three candidates are consistent with the observed pattern, and we set out each in turn together with the evidence bearing on it.

The first is depletion of the attentional resources that restoration presupposes. Attention Restoration Theory does not hold that a natural setting acts upon a passive recipient; it holds that restoration proceeds when soft fascination captures involuntary attention, permitting the directed-attention system to rest (31, 46). This mechanism requires that the person be able to disengage from effortful, goal-directed and threat-focused processing in the first place. Elevated stress compromises precisely this capability: acute and chronic stress impair prefrontal regulation of attention, reducing working memory capacity and the flexible reallocation of attention away from threat-relevant content (29). A highly stressed visitor may therefore register the forest as restorative - as the correlational data here indicate they do - while remaining unable to execute the attentional shift through which that restorative quality would ordinarily be realised. Review evidence is consistent with this reading: attentional restoration effects are detectable but modest, and the heterogeneity is greatest in samples whose baseline attentional state is neither measured nor reported (47).

The second is ruminative self-focus operating as a competing internal demand. Rumination is repetitive, self-referential and largely uncontrollable thought that occupies attention endogenously (41), and its prevalence rises with perceived stress. Where it is present, the environment must compete for attention against an internally generated stream that is both salient and self-sustaining. The experimental evidence here is instructive in both directions. A 90-min nature walk reduced self-reported rumination and subgenual prefrontal activation relative to an urban walk (40), which demonstrates that natural settings can interrupt the loop - but the exposure required to achieve this was substantially longer than the 20–30 min that defined eligibility in the present study. A dose sufficient to benefit a visitor who is not ruminating may be insufficient to interrupt an established ruminative cycle. On this account the attenuation observed here is in part a dose-threshold phenomenon: the restorative signal is not absent for highly stressed visitors, but the exposure obtained was too brief for it to displace the competing internal demand. This interpretation has the practical advantage of being directly actionable, and Section 5.4 builds on it.

The third is impaired emotion regulation. Stress Reduction Theory locates the initial restorative response in a rapid, largely pre-cognitive affective and psychophysiological shift on encountering an unthreatening natural setting (48). Converting that initial shift into a durable improvement in well-being nonetheless depends on regulatory processes: sustaining the positive affect once elicited, reappraising ongoing concerns in its light, and not immediately re-engaging the stressor (42). Chronic stress is associated with reduced reappraisal capacity and blunted positive-affect reactivity, so the initial response may well occur and then simply fail to consolidate. This mechanism sits most closely with the outcome measure used here, since the WHO-5 indexes well-being over the preceding 2 weeks rather than momentary state, and therefore captures exactly the consolidated form of benefit that a regulatory impairment would be expected to disrupt.

We cannot adjudicate among these three mechanisms with the present design, and we do not claim to. What the study can do is state what each of them predicts, so that the question is left in testable form rather than as an open appeal to plausibility. If attentional depletion is dominant, the moderation should reproduce when objective attentional measures such as backward digit span or Trail Making Test B are substituted for self-reported stress, and should be largest among visitors whose directed attention is most fatigued on arrival. If rumination is dominant, the moderation should attenuate as exposure duration increases beyond the 90-min threshold at which nature has been shown to interrupt self-referential processing, should be further moderated by trait rumination measured independently of perceived stress, and should weaken under protocols that actively interrupt self-referential thought, such as guided attention-anchoring walks. If regulatory impairment is dominant, the moderation should be visible in consolidated well-being outcomes but absent or reversed in momentary affect measured immediately after exposure - that is, highly stressed visitors would show the usual immediate affective response and then fail to retain it. These three predictions are mutually distinguishable within a single design that crosses varied exposure durations with both momentary and consolidated outcome measures, and we regard such a study as the most productive next step arising from the present findings.

The current findings also help clarify some inconsistencies in prior research. Although many systematic reviews find forest therapy to be beneficial, they note considerable heterogeneity across populations, settings and intervention designs (26). The heterogeneity of the studies may be explained by the fact that many researchers see stress as an outcome variable rather than a condition that directs the process of moderation. By directly incorporating stress as a moderator in our models, the present study explains why certain individuals or populations may benefit more from forest exposure than others. These factors may matter when comparing results across samples such as adolescents, students, clinicals and general park visitors with different baseline psychological conditions (49). The moderation model used here therefore clarifies the methodology and concept of a field that often uses direct effect assumptions.

It is worth being explicit about how the present result sits alongside the moderator analyses already available in this literature, since these constitute the most directly comparable prior work. Existing moderator analyses are almost exclusively environmental or dose-related in focus. Bolouki et al. (27) examine duration, nature type, sample characteristics and immersiveness; Song et al. (23) identify thermal conditions as modulating forest therapy outcomes; Kim et al. (9) test altitude; Kim et al. (22) test forest structure; Chu et al. (5) address acoustic and sensory integration. Each of these asks what property of the setting or of the dose changes the benefit obtained. The present study asks a different question - what property of the person changes it - and returns an answer of comparable magnitude to the environmental moderators reported in that work. The implication is that the heterogeneity those reviews document may be only partly attributable to differences between the settings compared, and partly attributable to unmeasured differences in the psychological state of the samples entering them, which is seldom reported and almost never modelled as a moderator. A concrete recommendation follows for that literature: baseline perceived stress should be reported as a standard sample descriptor in forest therapy and nature-prescription trials, and tested as a moderator wherever sample size permits, rather than being treated solely as an outcome.

### 5.3 Theoretical implications

The study also reveals that people often confuse actual environmental exposure with the perceived environmental quality. Recent studies on simulated nature and virtual nature suggest that even artificial nature can lead to well-being benefits, although this may depend on realism and immersion, sensory richness and interactivity, and user characteristics (24). Current evidence from a real national park indicates that perceptual evaluation is nevertheless a key component in ecologically valid environments. Forests confer a psychological well-being benefit, not merely through being-in-a-nature-experience, but whether the environment is cognitively registered as restorative at some level (Buxton et al., 2021). Using nature to cope with these stresses gives rise to new opportunities. In other words, the design of interventions should not just be about exposure, but also about how to promote positive perceptions of the environment through their sensory quality, safety, accessibility and interpretive engagement. Contrary to many of the studies conducted prior, which viewed forest environments as a homogeneous phenomenon, the current research looked at the influence of perceived environment on psychological well-being, taking into account perceived stress. Moreover, it incorporated an examination of various facets of forest environments, adding to a better understanding of the relation between particular aspects and psychological well-being.

The finding also carries a specific implication for the forest bathing literature. Umbrella reviews of shinrin-yoku report consistent benefit for mood and anxiety (19, 20), whereas meta-analyses employing standardised mood instruments recover smaller and more heterogeneous pooled effects (12, 21). If restorative benefit is conditional on the participant’s stress level in the manner observed here, then part of that discrepancy is expected rather than anomalous: pooled estimates will shift with the baseline stress composition of the samples entering them, which is rarely reported and therefore cannot currently be adjusted for. For the environmental perception literature (13, 30), the dimension-level correlations in Table 1 make a further point. Perceived restorative quality (*r* = 0.63) and tranquility (*r* = 0.59) carry appreciably more of the association with well-being than perceived biodiversity richness (*r* = 0.43) or safety (*r* = 0.46). Undifferentiated composite measures of environmental perception therefore average over attributes that behave differently, and disaggregated reporting should become the default where item counts permit.

### 5.4 Practical implications for National Parks

The study has a number of implications from a practical and public health perspective. Firstly, the findings promote the development of national parks as sites of preventive psychological well-being promotion. Forested parks like Zhangjiajie can be used as therapeutic landscapes which may help to improve emotional recovery and psychological state of people with low to moderate stress levels. Reflecting recent assertions for better integration of forest ecosystem services into wider health. The moderation finding carries a sharper implication than the general observation that provision should not be uniform. Because the association between environmental appraisal and well-being varied systematically with perceived stress, undifferentiated provision will predictably under-serve precisely those visitors with the greatest need. We therefore set out a three-tier scheme in which the intensity and type of intervention are matched to visitor stress level, with each tier anchored to a specific estimate from Section 4 rather than to general principle.

Tier 1 - low perceived stress (PSS-10 below approximately 12, corresponding to −1 SD; conditional slope *B* = 29.88). These visitors convert environmental appraisal into well-being most efficiently and require no programmatic intervention at all. The management objective for this tier is protection of appraisal quality rather than delivery of a service. Concretely, this means preserving the perceived attributes that carry most of the association in these data - quiet-zone designation and enforcement on the trails carrying the heaviest footfall, seating sited at points of high visual openness, and scheduling of maintenance, construction and motorised access outside peak visiting hours. Interventions at this tier are environmental rather than clinical, are inexpensive, and fall entirely within existing park authority competence.

Tier 2 - moderate perceived stress (PSS-10 approximately 12 to 25; conditional slope *B* = 21.50). Environmental appraisal remains substantially productive for these visitors, but less so than at Tier 1. The objective here is to add structure that lengthens and deepens engagement without requiring clinical resources: scheduled guided restorative walks of at least 60 to 90 min in place of unstructured visits; interpretive programming that directs attention specifically towards the attributes most strongly associated with well-being in these data, namely restorative quality (*r* = 0.63) and tranquility (*r* = 0.59), in preference to biodiversity signage (*r* = 0.43), which carries a weaker association; and repeat-visit mechanisms such as discounted season passes for local residents, since a single visit is unlikely to be the relevant dose. This tier is deliverable by existing park interpretation staff following short training and requires no new professional category.

Tier 3 - high perceived stress (PSS-10 above approximately 25, corresponding to +1 SD; conditional slope *B* = 13.12). This is the group for which unguided exposure should not be expected to suffice, and the appropriate provision is qualitatively different rather than merely more of the same. Three features distinguish it. First, duration: the evidence that nature exposure interrupts ruminative processing rests on exposures of around 90 min (40), well beyond the 20 to 30 min typical of the visits sampled here. Second, repetition rather than single contact. Third, protocols that actively support the attentional and regulatory processes that elevated stress compromises - attention-anchoring and mindfulness-based interpretation rather than free walking. Because delivery at this tier engages clinical competencies, it should be commissioned as a formal partnership between the park authority and local health services rather than run by the park alone, following the model established for nature prescriptions (24, 25). We note that this is also the tier least well evidenced by the present study, since visitors in acute distress were excluded at recruitment, and it should therefore be implemented with evaluation built in.

Operationalising the tiers does not require clinical screening at the park gate. The PSS-10 is a 10-item instrument that takes under 3 min to complete and can be embedded in an online booking flow or a visitor centre kiosk on an opt-in basis, returning a tier recommendation rather than a numerical score or any diagnostic label. Two safeguards are essential and we state them as conditions rather than suggestions. The instrument must be presented as a routing aid and not as a mental health assessment, with no diagnostic language and no retention of identifiable individual responses. And Tier 3 routing must terminate in a referral pathway agreed in advance with a named health provider, because identifying elevated distress without an onward route is worse than not screening at all. Where such a pathway cannot be established, parks should implement Tiers 1 and 2 only. These recommendations are offered as an evidence-informed design for evaluation rather than as validated practice, since the present study establishes the conditional association on which they rest but does not test the interventions themselves.

If perception matters, attribute park authority should concentrate on features that enhance restorative appraisal. These include visual quality, safety, tranquility, clarity of the trail, seating, biodiversity interpretation, and noise.

The innovation of this study is that we jointly analyzed forest environmental perception and stress moderation within the same analytical framework in a real-life national park situation. There is quite a bit of existing literature that demonstrates beneficial direct effects of forest exposure and fewer studies have examined the conditional effects. This study advances a more differentiated model of nature therapy, by showing that stress alters the psychological well-being benefit of positive perception of the natural environment.

### 5.5 Limitations and future research

The experiment was carried out on the visitors of national parks, who could be experiencing pleasant emotions due to tourism activities and vacation experiences. Thus, the results obtained regarding psychological well-being cannot completely depend on the forest environment, but can also be caused by restoration through tourism. Nonetheless, the study faces several limitations. The cross-sectional design does not allow for causal inference. While the observed associations make theoretical sense and are statistically significant, we cannot conclude the direction of causality. For instance, those with better psychological well-being may tend to evaluate the environment more positively. Next, the analysis looked at self-reported perceptual and psychological measures. While it is fitting for the study of subjective experience, the addition of a physiological marker, such as cortisol, heart rate variability or blood pressure, would strengthen the multidimensional assessment of therapeutic effect. The study was also limited to a single national park and might not be generalizable to other ecological, cultural, or management settings. Forest environments possess variability in the biodiversity, climates, accessibility and symbolic properties - which can change the perception-health relationships. Finally, the final regression model included no demographic variables. Although this was methodologically consistent with the analytic emphasis of the study, future research ought to be able to test whether age, gender, prior experience with a park or frequency of visits adds additional moderating variables to the patterns.

A further limitation concerns the temporal mismatch between the two central measures. The WHO-5 asks respondents about their well-being over the preceding 2 weeks, whereas forest environmental perception referred to the setting just experienced, whereas forest environmental perception was elicited in respect of the setting the respondent had just experienced. The two measures therefore refer to different time frames, and the temporal ordering runs contrary to the direction implied by the conceptual model: an appraisal formed on the day of the survey cannot have produced a well-being state that the instrument dates to the preceding fortnight. The observed association is accordingly more plausibly read as reflecting either a dispositional tendency for people in better psychological condition to appraise environments more favourably, or a common third factor such as trait affectivity influencing both reports, than as evidence of restoration occurring during the visit. The moderation finding is somewhat less exposed to this concern, since it concerns the strength of an association rather than its direction, but it too should be understood as describing a stable between-person pattern rather than a restorative process unfolding in real time. Capturing restoration itself requires momentary or pre-post measurement of affect rather than a two-week recall instrument, and we regard this as the single most important design change for future work in this area.

Four further limitations should be recorded. First, and as set out at length in Section 2.2, the sample was recruited by non-probability purposive intercept methods at a single park in a single month, so no estimate reported here supports design-based inference to any wider population; the exclusion of acutely distressed visitors additionally truncates the upper tail of the stress distribution, which means the moderation reported is likely to be conservative. Second, and relatedly, because eligibility screening and refusal were handled at the point of approach without a prospective tally, no response rate can be reported and the possibility that visitors who declined differed systematically from those who consented cannot be assessed; we regard prospective logging of approaches and refusals as a straightforward improvement that future studies of this design should adopt. Third, the forest environmental perception composite was adapted for this study and has not been validated against an independent sample or external criteria, so the psychometric evidence offered in Section 2.3 is internal to this dataset. Fourth, common method variance cannot be excluded. All three constructs were measured by self-report in a single administration, and although Harman’s single-factor test returned a first-factor loading well below the conventional threshold, that test is a weak diagnostic that can detect only gross method bias and cannot control for it (37). Designs incorporating temporally separated measurement, physiological indices, or informant reports would address this directly.

Future studies ought to go in a number of directions. Long-term and experimental models are required to aid in explaining whether repeated exposure to environmental perception or stress response is changed. Comparative studies of other types of forests, national parks and cultures would enable researchers to identify the environmental attributes that optimally support restorative perception. Future research must also aim to combine objective assessments of the environment and physiological biomarkers (e.g., heart rate, blood pressure) to current models of natural therapy with (subjective) perception information. Finally, intervention research is needed to validate or dismiss the idea that guided or customized park-based interventions can enhance psychological well-being outcomes in individuals with high levels of stress who seem to be relatively inert to positive environmental perception without intervention.

## 6 Conclusion

The current research investigated the relationship between perception of forest environment and psychological well-being in visitors of the Zhangjiajie National Forest Park, taking into account the moderating effect of perceived stress. Results have shown that more positive perceptions of forest environment were related to higher psychological well-being, while perceived stress was related to lower well-being. In addition, perceived stress was found to have a significant moderating effect on the mentioned relation. The association between favourable forest environmental perception and psychological well-being was weaker, though still positive, among visitors reporting higher perceived stress. Because the design is cross-sectional and the sample was recruited by non-probability intercept methods at a single park in a single season, these findings describe conditional associations within a defined visitor sample. They should be treated as hypotheses for experimental and longitudinal replication rather than as demonstrations of a therapeutic effect. Such results make an important contribution to the development of nature-based therapy studies because they show that responses to nature-based therapy depend on individual conditions.

## Data Availability

The original contributions presented in the study are included in the article, further inquiries can be directed to the corresponding author.
